# Predictors of growth hormone level on postoperative day one in patients with acromegaly

**DOI:** 10.1007/s12020-024-04130-6

**Published:** 2024-12-20

**Authors:** Haixiang Li, Ziqi Li, Tianshun Feng, Yuyang Chen, Jiansheng Zhong, Liangfeng Wei, Shousen Wang

**Affiliations:** 1 Department of Neurosurgery, Dongfang Affiliated Hospital of Xiamen University, School of Medicine, Xiamen University, Xiamen, China; 2 Fujian Provincial Clinical Medical Research Center for Minimally Invasive Diagnosis and Treatment of Neurovascular Diseases, Fuzhou, China; 3 Department of Neurosurgery, Fuzong Clinical Medical College of Fujian Medical University, Fuzhou, China; 4 Department of Neurosurgery, The 900th Hospital of Fuzhou, Fuzhou, China

**Keywords:** Acromegaly, Growth hormone, Predictors, Transsphenoidal surgery, Endocrine remission

## Abstract

**Purpose:**

The growth hormone (GH) level on postoperative day one (POD1), i.e., POD1GH, holds significant value in assessing surgical efficacy and predicting long-term remission in patients with acromegaly. This study aims to explore the factors that influence the GH level of POD1 after microscopic transsphenoidal surgery (mTSS) in patients with acromegaly, providing insights for preoperative clinical decisions.

**Methods:**

A total of 85 acromegaly patients undergoing mTSS were included in this study. Sex; age; body mass index (BMI); preoperative serum hormone levels and tumor characteristics were assessed for their correlation with POD1GH levels. POD1GH level non-remission, defined as POD1GH > 2.5 ng/mL, was considered an outcome.

**Results:**

The patients with acromegaly were divided into two groups: adult males (43 cases) and adult females (42 cases), with mean ages of 43.33 ± 11.92 years and 47.02 ± 14.18 years, respectively. Correlation and multivariate linear regression analyses revealed positive correlations of preoperative GH and prolactin (PRL) levels in females with POD1GH levels, while preoperative FT3 and TT levels in males were negatively correlated with POD1GH levels. Binary logistic regression and receiver operating characteristic (ROC) analyses identified preoperative GH levels ≥30.25 ng/mL (OR = 2.236, 95%CI = 1.402–3.567, *p* < 0.001), FT3 levels ≤4.415 pmol/L (OR = 0.329, 95%CI = 0.167–0.648, *p* < 0.001), and age ≤51 years (OR = 0.566, 95%CI = 0.352–0.911, *p* = 0.019) as independent risk factors for POD1GH level non-remission.

**Conclusions:**

Preoperative GH, FT3, TT, and PRL levels are correlated with POD1GH levels, with variations observed between sex. Age, preoperative GH, and FT3 levels can predict POD1GH level non-remission. Therefore, the comprehensive consideration of multiple hormone axes is necessary for predicting postoperative efficacy.

## Introduction

Acromegaly is a rare condition, with over 95% of cases attributed to pituitary adenoma (PA) secreting growth hormone (GH), leading to excessive levels of GH and insulin-like growth factor-1 (IGF-1) secretion [1]. Acromegaly is frequently accompanied by abnormalities in various anterior pituitary hormone levels and ultimately results in complications, such as hyperprolactinemia, hypogonadism, and central hypothyroidism, significantly affecting patients’ quality of life [2].

Transsphenoidal surgery (TSS) is an effective treatment modality for rapidly correcting patients’ endocrine disturbances. In clinical practice, however, some surgeons may still fail to achieve ideal postoperative GH levels despite employing more aggressive surgical approaches, even at the expense of impairing normal pituitary function or damaging surrounding tissues to achieve maximal tumor resection [3–5]. Early postoperative GH levels can determine whether patients with acromegaly can achieve long-term remission, including GH levels on postoperative day one (POD1) and day two, the mean of multiple GH measurements between 1 week and 3 weeks after neurosurgery, and nadir GH after an oral glucose tolerance test at least 7 weeks after neurosurgery, particularly the GH level on POD1 (i.e., POD1GH). Previous studies have indicated that a value of POD1GH > 2.5 ng/mL provides high predictive certainty for patients’ failure to achieve biochemical remission [6]. Therefore, investigating the predictors of POD1GH levels is of paramount importance for preoperative clinical decisions [7–10].

Current research primarily centers on predicting long-term remission, with limited investigation into the relationship between early postoperative growth hormone levels and preoperative factors, particularly hormonal levels, in patients with acromegaly [11, 12]. Nonetheless, perioperative hormone axis levels offer valuable insights into pituitary function and hold substantial potential for predicting the status of the growth hormone axis in the early postoperative phase [13].

The current study aims to explore the factors that influence POD1GH levels in acromegaly patients of different sex after microscopic transsphenoidal surgery (mTSS) and assist clinicians in choosing the optimal therapeutic strategy for their patients.

## Methods

### Patient cohort

Retrospective data collection included 130 patients with acromegaly who underwent mTSS at the Department of Neurosurgery, East Hospital of Xiamen University between 2011 and 2023. All surgical procedures were performed by the same neurosurgeon (S.W.), who had over 20 years of mTSS experience. Inclusion criteria were: 1) patients without prior radiation or surgical treatment; 2) patients with pathologically confirmed pituitary adenomas (PAs) that were immunohistochemically positive for growth hormone (GH), had serum GH and IGF-1 levels exceeding age- and sex-matched normal ranges, and showed intrasellar lesions on imaging; and 3) patients with complete surgical and serum hormonal examination data at various time points. The exclusion criteria were as follows: 1) patients aged ≤18 years, pregnant, or with diseases that interfere with GH/IGF-1 secretion (e.g., chronic kidney diseases, liver failure); 2) patients operated on by neurosurgeons other than S.W.; 3) patients with acromegaly treated with endoscopic TSS; 4) patients previously treated with medication (e.g., somatostatin analogs, dopamine receptor agonists); and 5) patients who experienced tumor recurrence or those who died. Finally, 85 patients who met the inclusion criteria formed the study cohort.

Ethical approval for all the procedures performed in this study was obtained from the Ethics Committee of the Dongfang Affiliated Hospital of Xiamen University. Written informed consent was obtained from all the participants.

### Radiological assessment

The pituitary was scanned using a 3.0-T magnetic resonance imaging (MRI) scanner (Tim Trio; Siemens Medical Solutions). Tumor characteristics were determined by two neurosurgeons and two radiologists on the basis of images, including tumor size, Knosp grade, and Hardy–Wilson classification. Tumor size was defined as the maximum diameter of PA measured using the INFINITT PACS system. PAs were categorized in accordance with their diameter, as follows: microadenomas (<1 cm), macroadenomas (1–4 cm), and giant adenomas (>4 cm). Knosp grade and Hardy–Wilson classification were used to classify PAs on the basis of cavernous sinus invasion, infrasellar invasion, and suprasellar extension on coronal MRI [14]. Patients underwent MRI examination within 3 months postoperatively to assess tumor resection extent. Gross-total resection (GTR) was defined as 100% tumor removal on postoperative MRI, while subtotal resection (STR) was defined as 90–100% tumor removal [15].

### Endocrinological assessment

Serum IGF-1 and hormone levels, including GH, free triiodothyronine (FT3), free thyroxine (FT4), total triiodothyronine (TT3), total thyroxine (TT4), thyroid-stimulating hormone (TSH), prolactin (PRL), estradiol (E2), total testosterone (TT), follicle-stimulating hormone (FSH), luteinizing hormone (LH), cortisol (COR), and adrenocorticotropic hormone (ACTH), were recorded within 3 days preoperatively. POD1GH was recorded within 1 day postoperatively. All hormone samples were obtained at 8 a.m. on an empty stomach and analyzed using the chemiluminescence method with Siemens ADVIA Centaur XP machine.

POD1GH level non-remission was defined as POD1GH level >2.5 ng/mL, while POD1GH level remission was defined as POD1GH level ≤2.5 ng/mL. IGF-1 levels were expressed as the ratio of the baseline value (ng/mL) to the upper limit of normal (ULN) IGF-1 value adjusted for age and sex.

### Data analysis

Data analysis was conducted using IBM SPSS 25.0 (IBM Corporation) for statistical analysis, and GraphPad Prism (version 9.5.0) was used for visualization and presentation of graphical data. Continuous variables were presented as mean ± standard deviation or median (interquartile range) based on data distribution, while categorical variables were expressed as numbers (percentages). Statistical comparisons were made using the independent sample t-test for normally distributed continuous variables, the Mann–Whitney U-test for non-normally distributed continuous and ordinal categorical variables, and the chi-squared test or Fisher’s exact test for unordered categorical variables. These values were log-transformed using base-10 due to the skewed distribution of POD1GH levels. Pearson correlation analysis was conducted to assess correlations between continuous normally distributed variables, while Spearman correlation analysis was employed for non-normally distributed variables. Multiple linear regression analysis was performed to evaluate the independent relationships between hormone and POD1GH levels in male and female patients separately, adjusting for potential confounding factors by using stepwise regression. Binary logistic regression analysis was conducted to determine independent risk factors for POD1GH non-remission, and the effectiveness of each independent factor in predicting POD1GH non-remission was compared using the area under the receiver operating characteristic (ROC) curve (AUC). The Youden index was calculated to determine the optimal cutoff value for distinguishing POD1GH non-remission based on hormone levels. A *p*-value of <0.05 was considered statistically significant.

## Results

### Clinical data

This study included 85 patients with a mean age of 45.15 ± 13.14 years (range: 19–72 years). They were divided into the adult male group (43 cases), with a mean age of 43.33 ± 11.92 years (range: 19–72 years), and the adult female group (42 cases), with a mean age of 47.02 ± 14.18 years (range: 20–70 years). No significant differences in age or body mass index were found between the two groups (Table 1).

**Table 1:** Baseline characteristics in patients with acromagely

Characteristics	Male (*n* = 43)	Female (*n* = 42)	*p*-value
Age, years	43.33 ± 11.92	47.02 ± 14.18	0.196
BMI, kg/m^2^	25.36 ± 2.50	24.73 ± 3.36	0.336
Tumor size, mm	20.17 ± 9.80	22.63 ± 10.36	0.265
Tumor size, *n* (%)			0.999
Microadenomas (<1 cm)	4 (9.30%)	4 (9.52%)	
Macroadenomas (1–4 cm)	37 (86.05%)	36 (85.71%)	
Giant adenomas (≥4 cm)	2 (4.65%)	2 (4.77%)	
Knosp grade, *n* (%)			0.175
0	10 (23.26%)	9 (21.43%)	
I	11 (25.58%)	7 (16.67%)	
II	6 (13.95%)	2 (4.77%)	
IIIA	4 (9.30%)	7 (16.67%)	
IIIB	5 (11.63%)	5 (11.90%)	
IV	7 (16.28%)	12 (28.56%)	
HW grade, *n* (%)			0.772
0	5 (11.63%)	3 (7.14%)	
I	4 (9.30%)	2 (4.77%)	
II	15 (34.88%)	13 (30.95%)	
III	8 (18.60%)	10 (23.81%)	
IV	11 (25.58%)	14 (33.33%)	
HW stage, *n* (%)			0.187
A	30 (69.77%)	22 (52.38%)	
B	5 (11.63%)	5 (11.91%)	
C	8 (18.60%)	15 (35.71%)	
D	0 (0%)	0 (0%)	
E	0 (0%)	0 (0%)	
Pathology, *n* (%)			0.238
GH	31 (72.09%)	23 (54.76%)	
GH + PRL	10 (23.26%)	16 (38.10%)	
GH + (≥2 hormones)	2 (4.65%)	3 (7.14%)	
POD1GH level remission, *n* (%)			0.296
Yes	25 (58.14%)	29 (69.05%)	
No	18 (41.86%)	13 (30.95%)	

Values are shown as count (percentage) or mean ± SD

*HW* Hardy-Wilson

According to their medical history, 34 patients (40%) experienced headaches, 18 (21%) suffered from dizziness, 49 (58%) experienced visual impairment, and 62 (73%) exhibited facial and physical changes, including enlargement of the limb joints and fingertips, thickened lips, macroglossia, widened nasal bridge, protruding forehead, and deepening of voice. In addition, 26 (31%) had hypertension, 52 (61%) had dyslipidemia, 29 (34%) had diabetes mellitus, 43 (51%) had structural or functional cardiac abnormalities based on echocardiography, and 40 (48%) had nodular goiter based on thyroid ultrasound. According to surgical and imaging data, gross total resection (GTR) was achieved in 75 cases (88.24%) and subtotal resection (STR) in 10 cases (11.76%). Intraoperative cerebrospinal fluid leakage occurred in 21 cases (25%), with artificial dura mater patches used for repair in 16 of these cases (19%). Pituitary hemorrhage occurred in 7 cases (8%), and infarction in 1 case (1%). No cases (0%) of postoperative cerebrospinal fluid leakage were reported, but hyponatremia occurred in 18 cases (21%), central diabetes insipidus in 43 cases (51%), central nervous system infection in 1 case (1%), and sinusitis in 1 case (1%).

On the basis of radiological data, the mean tumor maximum diameter in the cohort was 21.39 ± 10.09 mm. In particular, 8 cases (9.41%) were microadenomas, 73 (84.71%) were macroadenomas, and 4 (4.71%) were giant adenomas. No significant differences were recorded between the 2 groups in terms of tumor size, Knosp grade, Hardy–Wilson grade, or Hardy–Wilson stage.

Immunohistochemical staining results showed that 54 patients (63.53%) exhibited positive staining for GH only, 26 (30.59%) showed positive staining for both GH and PRL, and 5 (5.88%) demonstrated positive staining for GH and multiple (≥2) hormones. No significant differences occurred in the immunohistochemical staining results between the two groups.

### Preoperative and postoperative hormone level changes

In this study cohort, a significant decrease in GH levels was observed postoperatively (p < 0.001). Among them, 54 patients achieved the remission criteria for POD1GH levels, while 31 patients did not, with no statistically significant difference in the proportion of remission between sex (p = 0.296). Preoperative IGF-1 level data were available for 69 patients (81.18%), including 34 males (50.77%) and 35 females (49.23%), with no significant difference in preoperative IGF-1 levels between the two groups (p = 0.288).

**Fig. 1:**
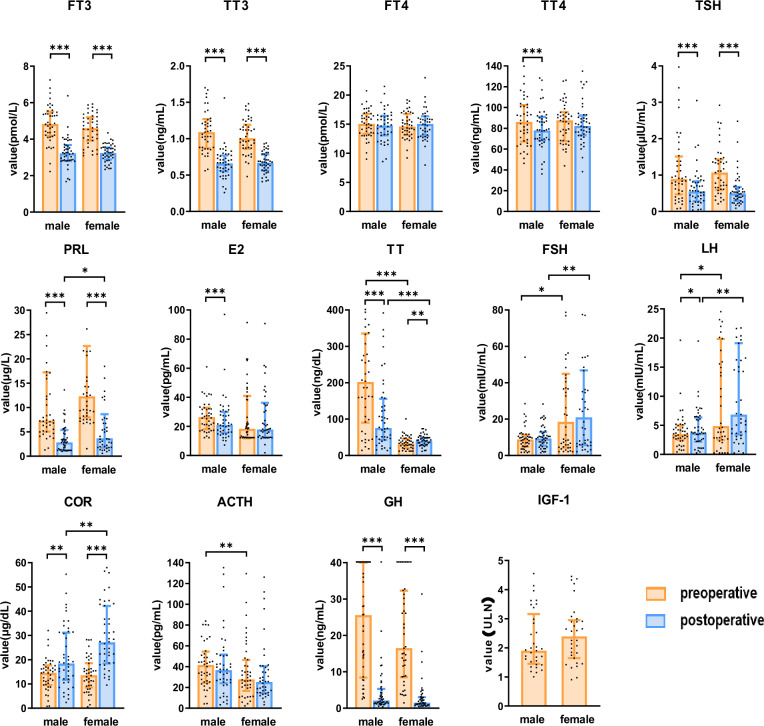
The details of perioperative and postoperative hormone levels in male and female patients with acromegaly. *: p < 0.05; **: p < 0.01; ***: p < 0.001

Postoperatively, FT3 (p < 0.001), TT3 (p < 0.001), TSH (p < 0.001), and TT4 (p < 0.001) levels significantly decreased in male patients with acromegaly, while no significant changes were observed in FT4 (p = 0.171; p = 0.416) levels in both sexes and TT4 (p = 0.970) levels in females. COR (p = 0.005; p < 0.001) levels significantly increased postoperatively, while no significant change was observed in ACTH (p = 0.726; p = 0.945) levels (Table 2) (Fig. 1).

**Table 2:** Preoperative and postoperative hormones

Characteristics	Male (*n* = 43)		*p*-value	Female (*n* = 42)		*p*-value
	Before surgery	After surgery		Before surgery	After surgery	
GH, μg/L	25.60(8.43–40)	2.13(1.2–5.29)	<0.001	16.50(8.52–32.3)	1.51(0.74–3.05)	<0.001
IGF-1, ULN	1.89(1.40–3.06)	-		2.37(1.64–2.95)	-	
FT3, pmol/L	4.83(4.18–5.51)	3.22(2.8–3.68)	<0.001	4.60(3.92–5.20)	3.24(2.88–3.54)	<0.001
TT3, ng/mL	1.09(0.87–1.27)	0.66(0.55–0.77)	<0.001	1.01(0.86–1.19)	0.67(0.56–0.79)	<0.001
FT4, pmol/L	15.05(13.35–16.77)	14.74(13.08–16.42)	0.171	14.59(13.12–16.76)	15.05(12.98–16.36)	0.416
TT4, ng/mL	86.10(67.3–102.4)	78.00(68.5–91.0)	<0.001	87.65(67.6–95.55)	82.2(73.45–92.33)	0.970
TSH, μIU/mL	0.89(0.47–1.51)	0.55(0.26–0.83)	<0.001	1.07(0.59–1.43)	0.49(0.30–0.68)	<0.001
PRL, ug/L	7.29(5.05–17.22)	2.81(1.16–5.40)	<0.001	12.30(12.30–22.66)	3.69(1.78–8.64)	<0.001
E2, pg/mL	26.36(18.07–32.81)	20.95(15.49–29.88)	<0.001	18.34(11.8–40.95)	17.84(12.33–36.28)	0.577
TT, ng/dL	202.19(90.19–335.5)	75.61(42.71–156.05)	<0.001	32.98(20.05–45.54)	40.23(28.2–48.63)	0.018
FSH, mIU/mL	8.78(4.82–11.34)	9.30(5.39–12.91)	0.391	18.52(4.32–44.95)	21(5.76–46.76)	0.412
LH, mIU/mL	3.46(2.54–4.98)	3.69(2.19–6.35)	0.021	4.88(2.50–19.83)	6.83(3.41–19.12)	0.051
COR, μg/dL	14.55(9.52–17.65)	18.43(11.68–31.13)	0.005	13.60(8.53–18.58)	27.16(18.41–42.14)	<0.001
ACTH, pg/mL	41.50(24.59–54.42)	36.64(24.04–51.51)	0.726	27.65(16.68–46.43)	25.09(14.43–40.40)	0.945

Data are presented as the median (interquartile range)

Postoperatively, PRL levels decreased significantly in patients with acromegaly (p < 0.001), with no significant changes observed in FSH (p = 0.391; p = 0.412) levels, while LH levels increased significantly (p = 0.021; p = 0.051). Compared with the male patients, the female patients had significantly higher preoperative levels of PRL (p = 0.017), FSH (p = 0.034), and LH (p = 0.046), and lower levels of E2 preoperatively (p = 0.053) and postoperatively (p = 0.806), although these differences were not statistically significant. No significant differences were found in E2 levels preoperatively and postoperatively in females (p = 0.577), but postoperative E2 levels significantly decreased in male patients (p < 0.001). Postoperatively, TT levels significantly decreased in male patients (p < 0.001), but significantly increased in female patients (p = 0.018).

### Correlation of tumor diameter, Knosp grade, and Hardy–Wilson classification with preoperative hormones

**Fig. 2:**
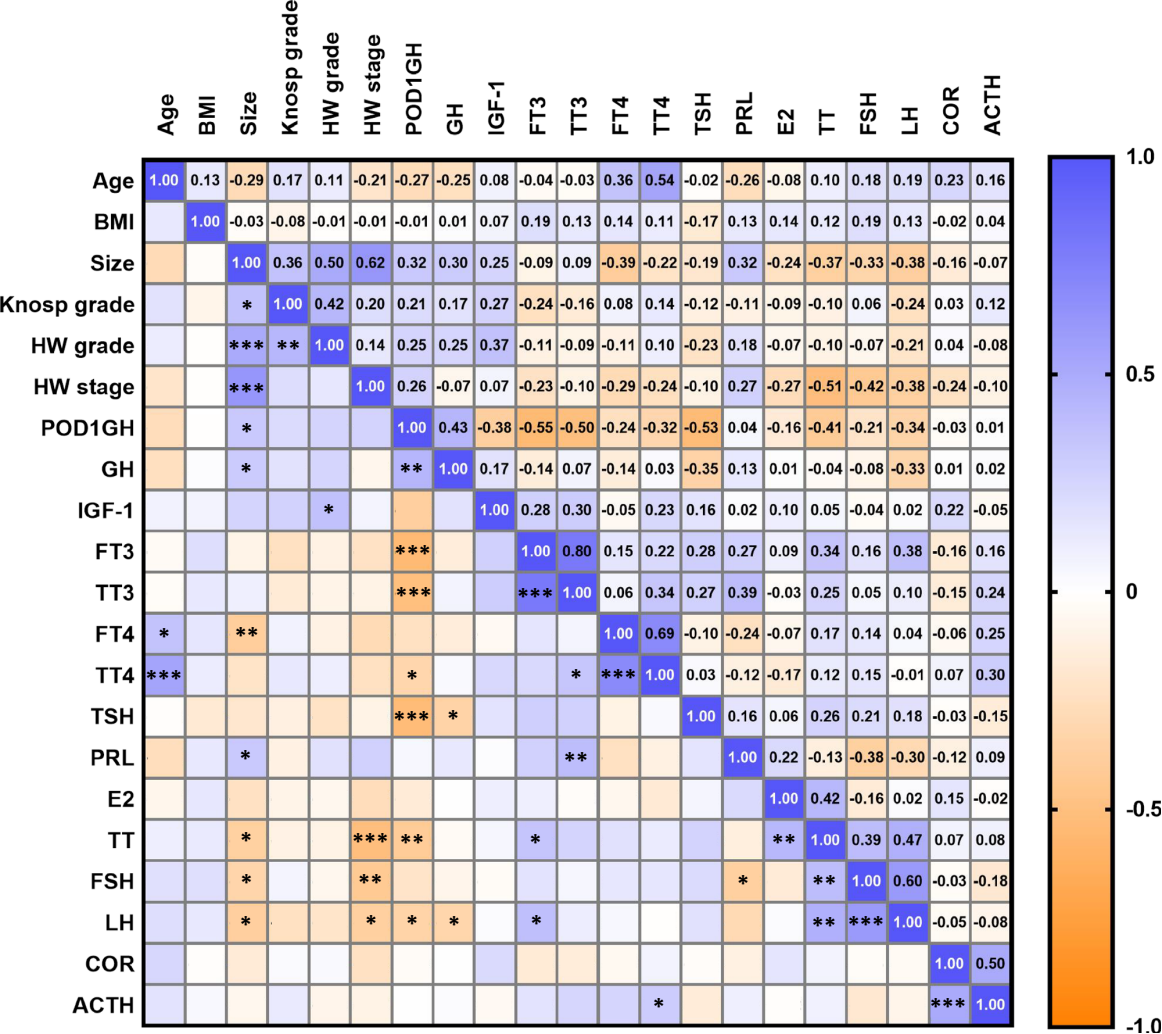
The correlations between the preoperative factors and the POD1GH level in male patients with acromegaly. *: p < 0.05; **: p < 0.01; ***: p < 0.001

In male patients with acromegaly, tumor maximum diameter was negatively correlated with preoperative FT4 (p = 0.010), FSH (p = 0.030), LH (p = 0.012) and TT (p = 0.015) levels. In addition, positive correlations were found between tumor maximum diameter and preoperative GH (p = 0.047) and PRL (p = 0.037) levels. Hardy–Wilson stage was negatively correlated with preoperative FSH (p = 0.005), LH (p = 0.013), and TT (p < 0.001) (Fig. 2).

**Fig. 3:**
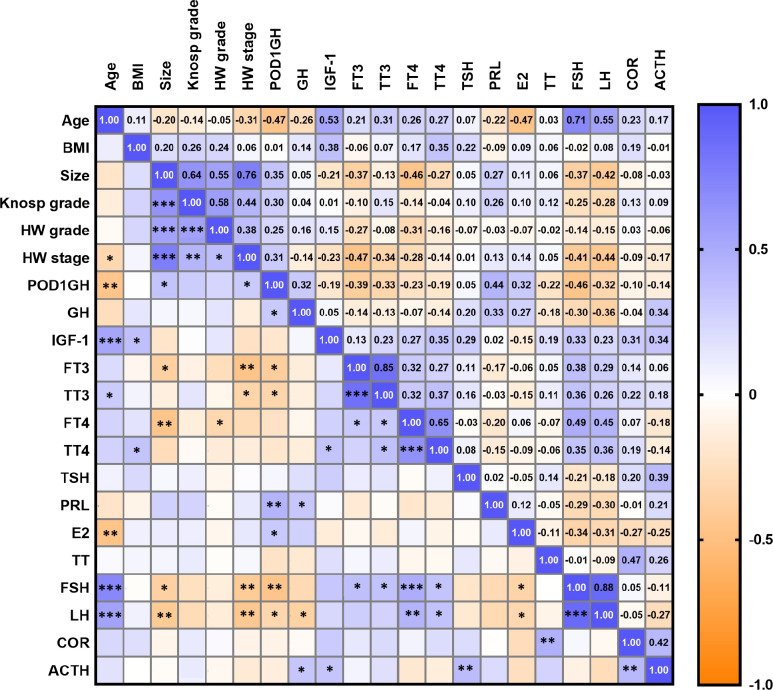
The correlations between the preoperative factors and the POD1GH level in female patients with acromegaly. *: p < 0.05; **: p < 0.01; ***: p < 0.001

In female patients with acromegaly, tumor maximum diameter was negatively correlated with preoperative FT4 (p = 0.002), FSH (p = 0.017), LH (p = 0.006), and FT3 (p = 0.017) levels. Hardy–Wilson stage was negatively correlated with preoperative FSH (p = 0.007), LH (p = 0.004) (Fig. 3).

No correlation was observed between preoperative hormone levels and Knosp grade in both patient groups.

### Correlation between preoperative hormones

Preoperative GH was negatively correlated with LH (p = 0.032; p = 0.019) levels, but not correlated with FSH, FT3, FT4, TT3, TT4, COR, ACTH, TT, and E2 levels.

### Correlation among age, preoperative hormones, tumor characteristics, and POD1GH levels

Patients with non-remission POD1GH levels were younger (p = 0.003) and had larger tumor diameter (p < 0.001), higher GH (p < 0.001), and lower FT3 (p < 0.001), TT3 (p = 0.004), TSH (p = 0.002), FSH (p < 0.001), and LH (p = 0.002) levels (Table 3).

**Table 3:** POD1GH level remission and clinical correlations

Variable	Remission After Surgery (*n* = 54)	Nonremission After Surgery (*n* = 31)	*p*-value
Age, years	48.35 ± 13.19	39.58 ± 11.20	0.003
BMI, kg/m^2^	24.90 ± 2.96	25.31 ± 3.00	0.540
Tumor size, mm	18.78 ± 7.50	25.95 ± 12.32	<0.001
Tumor size, *n* (%)			0.009
Microadenomas (<1 cm)	7(12.96%)	1(3.23%)	
Macroadenomas (1–4 cm)	47(87.04%)	26(83.87%)	
Giant adenomas (≥4 cm)	0	4(12.90%)	
Sex, *n* (%)			0.296
Male	25 (46.3%)	18(58.07%)	
Female	29(53.7%)	13 (41.93%)	
Knosp grade, *n* (%)			0.186
0	15(27.78%)	4(12.90%)	
I	11(20.37%)	7(22.58%)	
II	5(9.26%)	3(9.68%)	
IIIA	7(12.96%)	4(12.90%)	
IIIB	8(14.81%)	2(6.45%)	
IV	8(14.81%)	11(35.48%)	
HW grade, *n* (%)			0.266
0	7(12.96%)	1(3.23%)	
I	4(7.41%)	2(6.45%)	
II	20(37.04%)	8(25.8%)	
III	11(20.37%)	7(22.58%)	
IV	12(22.22%)	13(41.94%)	
HW stage, *n* (%)			0.082
A	38(70.37%)	14(45.16%)	
B	4(7.41%)	6(19.35%)	
C	12(22.22%)	11(35.48%)	
D	0(0%)	0(0%)	
E	0(0%)	0(0%)	
Preoperative hormone level			
GH, μg/L	14.50(6.86–28.05)	40.00(17.40–40.00)	<0.001
IGF-1, ULN	2.23(1.59–3.20)	1.81(1.27–2.52)	0.060
FT3, pmol/L	4.94(4.43–5.53)	4.13(3.52–5.08)	<0.001
TT3, ng/mL	1.12(0.95–1.27)	0.94(0.77–1.13)	0.004
FT4, pmol/L	14.95(13.75–16.85)	14.14(12.88–16.33)	0.089
TT4, ng/mL	90.72(77.20–100.48)	75.30(62.20–100.50)	0.053
TSH, uIU/mL	1.09(0.74–1.61)	0.59(0.30–1.32)	0.002
PRL, μg/L	8.55(6.98–13.80)	15.80(6.18–33.00)	0.122
E2, pg/mL	19.76(12.76–33.25)	25.43(16.87–40.19)	0.176
TT, ng/dL	52.39(32.50–229.96)	46.06(22.02–184.52)	0.376
FSH, mIU/mL	10.50(6.70–36.39)	5.96(2.20–11.27)	<0.001
LH, mIU/mL	4.65(2.91–14.98)	2.76(1.2–4.12)	0.002
COR, μg/dL	14.26(10.41–18.64)	12.62(7.12–16.28)	0.218
ACTH, pg/mL	31.82(21.50–48.00)	33.81(20.64–51.62)	0.942

Values are presented as the mean ± standard deviation, number (%), or median (interquartile range), according to the distribution of data, unless indicated otherwise

*HW* Hardy-Wilson

Correlation analysis showed that in male patients, POD1GH levels were positively correlated with size (p = 0.037) and GH (p = 0.004) levels, but negatively correlated with age (p = 0.081) and FT3 (p < 0.001), TT3 (p < 0.001), TT4 (p = 0.034), TSH (p < 0.001), TT (p = 0.007), and LH (p = 0.024) levels. The correlation coefficient (r) between POD1GH levels and TT4 levels (r = −0.324) was considerably lower than those between POD1GH levels and FT3 (r = −0.549), TT3 (r = −0.503), and TSH (r = −0.531) levels. In female patients, POD1GH levels were positively correlated with size (p = 0.023), Hardy–Wilson stage (p = 0.045), GH (p = 0.040), PRL (p = 0.003), and E2 (p = 0.038) levels, but negatively correlated with age (p = 0.002), FT3 (p = 0.012), TT3 (p = 0.034), FSH (p = 0.002), and LH (p = 0.040) levels.

**Fig. 4:**
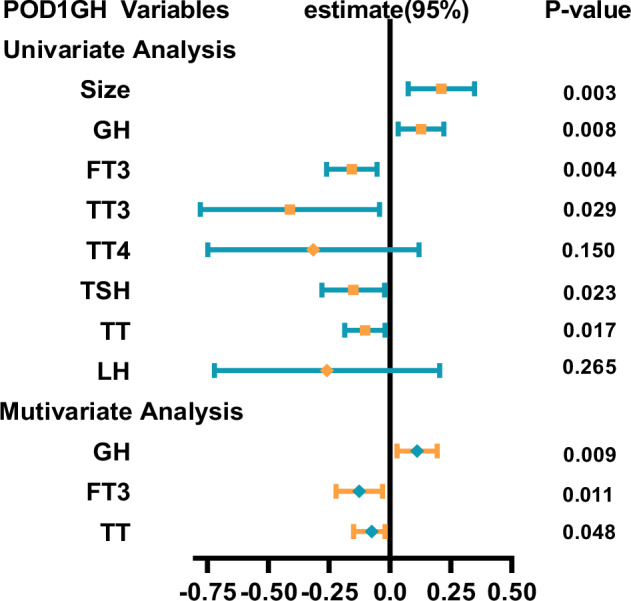
The linear regression analyses of POD1GH level in male patients with acromegaly

**Fig. 5:**
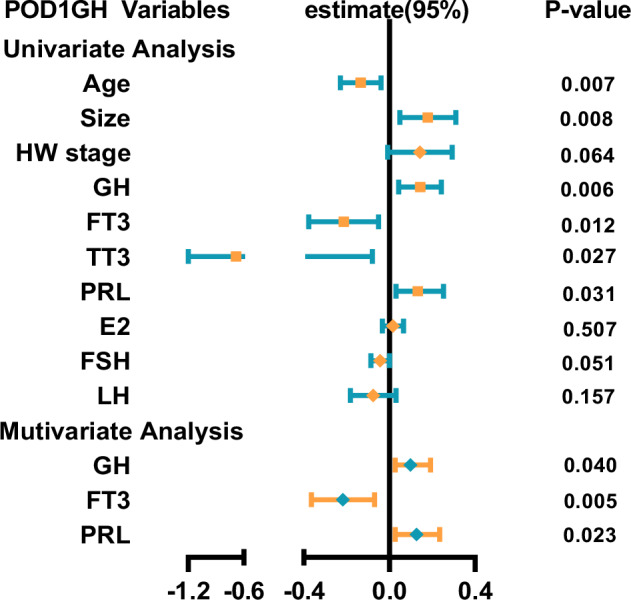
The linear regression analyses of POD1GH level in female patients with acromegaly

Multiple linear regression analysis revealed that size and preoperative GH, FT3, TT3, TSH, and TT were independent predictors of POD1GH levels in male patients (Fig. 4), while age, size, and preoperative GH, FT3, TT3, and PRL were independent predictors of POD1GH levels in female patients (Fig. 5). Multifactorial analysis indicated that preoperative GH (β = −0.149; 95% confidence interval [CI] = − 0.075, −0.01; p = 0.009), FT3 (β = −0.126; 95%CI = − 0.221, −0.030; p = 0.011), and TT (β = −0.075; 95%CI = − 0.149, −0.001; p = 0.048) were independent predictors of POD1GH levels in male patients, with higher preoperative GH levels or lower FT3 and TT levels associated with higher POD1GH levels in male patients. Preoperative GH (β = 0.005; 95%CI = 0.098, 0.019; p = 0.040), FT3 (β = −0.346; 95%CI = − 0.217, −0.070; p = 0.005), and PRL (β = 0.126; 95%CI = 0.018, 0.234; p = 0.023) levels were independent predictors of POD1GH levels in female patients, with higher preoperative GH levels or lower FT3 and PRL levels associated with higher POD1GH levels in female patients.

**Fig. 6:**
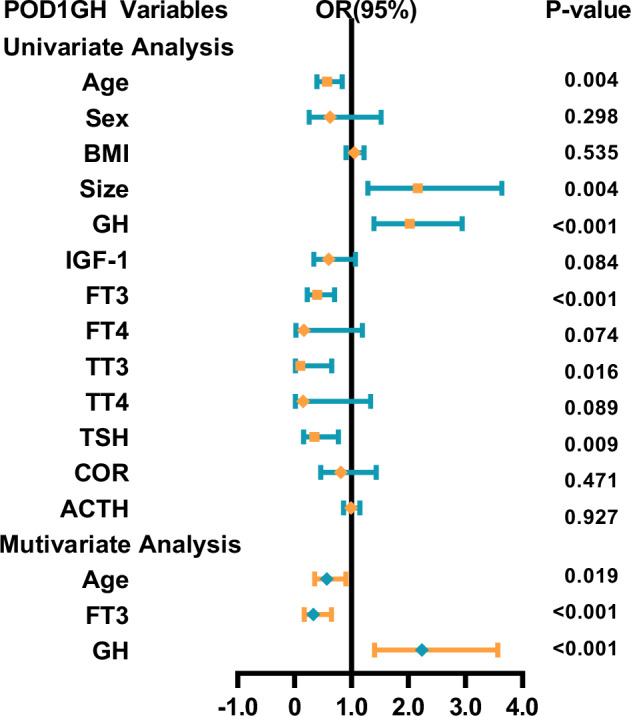
The logistic regression analyses of POD1GH level in patients with acromegaly

**Fig. 7:**
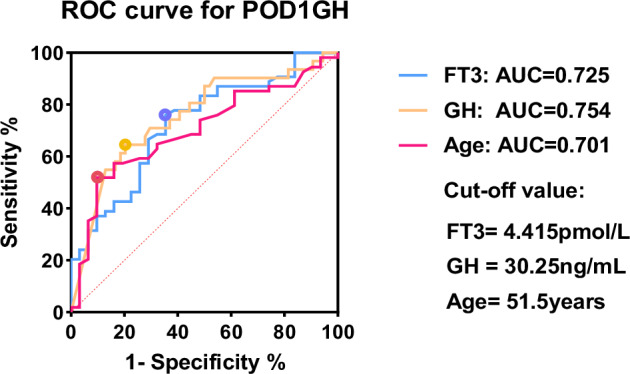
Performance of ROC curve analysis for predicting POD1GH level non-remission

Some acromegaly patients may experience delayed remission after surgery. To maximize specificity in identifying cases without remission, this study used a cut-off value of 2.5 ng/mL to distinguish between remission and non-remission, followed by binary logistic regression analysis. The univariate analysis results showed that age, size, and preoperative GH, FT3, TT3, FSH, and LH levels were predictors of POD1GH level non-remission (Fig. 6). The multivariate analysis results revealed that preoperative GH (OR = 2.236, 95%CI = 1.402–3.567, p < 0.001) and FT3 (OR = 0.329, 95%CI = 0.167–0.648, p < 0.001) levels, and age (OR = 0.566, 95%CI = 0.352–0.911, p = 0.019) were independent predictors of POD1GH level non-remission. The ROC analysis (Fig. 7) demonstrated that preoperative GH levels ≥30.25 ng/mL (AUC = 0.754), FT3 levels ≤4.415 pmol/L (AUC = 0.725), and age ≤51 years (AUC = 0.701) can predict POD1GH level non-remission.

## Discussion

### Preoperative and postoperative hormonal level changes

Previous studies research indicates that GH can lead to decreased FT4 secretion [16], consistent with our result. While no significant postoperative changes in FT4 were observed in our study, this may be explained by the combined effect of a substantial postoperative decrease in GH that promotes increased FT4 secretion and intraoperative manipulation of the pituitary or the associated stress response, both of which have been shown in previous studies to suppress FT4 secretion or even cause pituitary insufficiency [17–19]. For the HPA axis, in our opinion, stress-induced ACTH secretion during the perioperative period typically leads to an increase in ACTH levels. However, postoperative pituitary insufficiency causes a decrease in ACTH levels after surgery, as reported [20]. Consequently, postoperative ACTH levels differ significantly from preoperative levels, as observed in our study. The preoperative GH levels were also significantly correlated with anterior pituitary hormones, particularly LH, several studies have suggested that high GH levels are among the reasons for decreased LH secretion in patients with acromegaly [21–23]. The lack of correlation with FSH in our results may imply that the relationship between LH and GH is closer than that between FSH and GH.

Regarding the PRL and HPG axes, our study demonstrates that total testosterone plays a pivotal role in regulating sex hormones in male patients with acromegaly. The factors contributing to change in TT secretion levels are as follows: Postoperative decrease in PRL is insufficient to positively affect TT levels by alleviating gonadotropin suppression. Additionally, stress-induced cortisol secretion further contributes to significant reduction in TT levels, as evidenced by the marked elevation of COR observed in our results.

PRL levels in females are known to be relatively higher than in males. It is interesting to speculate that PRL dominates over other sex hormones and inhibits gonadotropins in female patients with acromegaly, leading to the preoperative decrease in E2 and TT levels. The increase in postoperative TT secretion is likely due to the significant postoperative decline in PRL, which alleviates gonadotropin inhibition, resulting in increased LH secretion and ultimately enhanced TT production, as indicated by the marked elevation of LH observed in our results.

Our results demonstrate that in male patients with acromegaly, larger tumor diameters are associated with higher levels of GH and PRL, consistent with previous findings, potentially due to the pituitary stalk effect and hormonal co-secretion [24, 25]. Additionally, we found tumor diameter and Hardy–Wilson grade were associated with reduced levels of FT4, FSH and LH in patients of both sexes. Tumor invasion of the hypothalamus and pituitary gland may be the underlying causes of these endocrine disturbances.

### Relationship between preoperative hormone levels and POD1GH levels

The two major thyroid hormones in circulation are thyroxine and triiodothyronine, with FT4 converted to its more active form FT3 by three deiodinases [26]. The literature suggests that preexisting hypothyroidism increases the risk of not achieving long-term biochemical remission postoperatively [11]. This study further demonstrates that in patients with acromegaly, POD1GH levels are not correlated with FT4, and ultimately, FT3 instead of FT4 is established as an independent risk factor for POD1GH level non-remission. This finding indicates that FT3 is more valuable than FT4 in predicting POD1GH levels, possibly due to the considerably higher activity of FT3 in circulation than of FT4, allowing it to sensitively reflect recent somatotropic axis functional status [17, 27–30]. Therefore, FT3 is important for predicting postoperative somatotropic axis function, while FT4 is essential for preoperative assessment of thyroid function status. GH can increase the conversion of TT4 to TT3, and TT4 significantly decreases when the disease progresses to a certain extent, decreasing TT3 and FT3 levels. In case of thyroid dysfunction, FT4 decreases before FT3, with lower specificity in predicting non-remission risk. In acromegaly patients with a typically prolonged disease course, a significant decrease in FT3 may indicate poorer prognosis [19]. TT3 and TT4 lack predictive values due to their susceptibility to certain factors, such as thyroid-binding globulin and albumin. Therefore, FT3 is a more accurate and feasible indicator than FT4, TT3, and TT4 for reflecting POD1GH levels. Thyrotrophs account for only about 5% of the anterior pituitary, and TSH does not predict POD1GH levels. Conversely, GH and PRL are secreted by eosinophilic cells, which are the most abundant cells in the anterior pituitary, making them representative of somatotropic axis function [31].

GH receptors are widely distributed in the reproductive tract, gonadotropin-releasing hormone neurons, and pituitary gonadotrophin cells. Sex hormones can modulate the somatotropic axis, participating in the regulation of GH-releasing hormone secretion. Concurrently, GH can reciprocally regulate the levels of sex hormones by acting on interstitial cell [32, 33]. Reports suggest that tumor compression and excess GH in the blood can affect FSH/LH secretion, leading to decreased TT levels [25, 34, 35], and TT significantly influences the somatotropic axis [36]. The current study found that lower preoperative TT levels in males predict higher GH levels on POD1, supporting previous findings and suggesting that TT levels may directly or indirectly reflect the functional state of the growth hormone axis. Recent research indicates that normal PRL levels at diagnosis can serve as an independent predictor of long-term remission [2, 37]. Our results indicate that elevated preoperative PRL levels in females predict higher GH levels on POD1. This suggests that preoperative PRL levels are valuable not only for forecasting biochemical outcomes but also for assessing early postoperative somatotropic axis function. In summary, preoperative measurement of TT and PRL levels may provide an additional metric for clinicians to assess disease severity and postoperative prognosis.

Preoperative growth hormone GH levels have long been recognized as a predictor of remission [4, 10, 38–40]. In contrast to earlier studies, this research employed postoperative day 1 POD1GH levels as a reference to detect early changes in GH levels and suggest that preoperative GH levels ≥30.25 ng/mL are associated with a reduced likelihood of achieving immediate biochemical remission postoperatively. Such patients may need to adjust their expectations and prepare for potential adjunctive therapies in the future. However, despite evidence from several studies linking preoperative IGF-1 levels with final remission rates, our study found no correlation between preoperative IGF-1 levels and POD1GH levels. This discrepancy may arise because IGF-1 levels are more indicative of long-term disease outcomes and exhibit substantial individual variability influenced by factors such as age, sex, and metabolic state. Current research conditions and data are inadequate to effectively standardize these measures, preventing the establishment of a robust predictive model using IGF-1. Therefore, further validation through studies with larger sample sizes is necessary.

### Relationship among age, tumor characteristics, and POD1GH levels

Numerous studies have investigated the use of age to predict biochemical remission in patients with acromegaly and found that older patients generally exhibit lower GH secretion levels compared to the general population [11, 41, 42]. Our data set indicated patients over the age of 51 often experience a decrease in GH levels to age-appropriate low ranges shortly after tumor resection, which increases the likelihood of achieving long-term remission. Although the correlation analysis results were consistent with those of previous studies, indicating an association between tumor maximum diameter and postoperative GH levels [2–4, 9, 39, 40], our regression analysis did not observe predictive values of tumor diameter, Knosp grade, and Hardy–Wilson classification for POD1GH levels. This finding suggests that hormones may serve as an adjunct to imaging studies, aiding in the assessment of early postoperative somatotropic axis function, especially when imaging findings are inconclusive, consistent with the views of Sarkar et al. [41]. Preoperative and postoperative hormonal monitoring are routine tests for hospitalized patients, and they exhibit high accuracy and simplicity. Requiring GH/IGF-1 level monitoring at 3 months or 6 months postoperatively necessitates high patient compliance. The use of POD1GH levels can compensate for this deficiency, aiding in the rough evaluation of surgical efficacy early on and the formulation of subsequent treatment plans to accelerate disease recovery.

In clinical practice, even if patients achieve complete tumor resection, postoperative GH levels may still not decrease to the desired range [39, 40]. In such cases, considering further radiotherapy or pharmacological treatment is necessary. Previous research indicates that patients requiring additional postoperative treatment typically have higher POD1GH levels than those who do not [43]. Therefore, the preoperative prediction of postoperative GH levels is significant, not only to reduce doctor–patient conflicts but also to assist in selecting preoperative surgical plans and adjusting intraoperative surgical plans. In cases of severe tumor invasion into the cavernous sinus, where predicted POD1GH levels may not decrease to the ideal range, a more conservative surgical approach may be considered, supplemented with medication and radiotherapy for subsequent treatment [44, 45]. Conversely, when conditions permit, maximizing the reduction of postoperative GH levels could be a favorable option for clinicians, as it helps to correct endocrine dysfunction to the greatest extent and may reduce the risk of tumor recurrence [5].

## Conclusions

For patients with acromegaly, preoperative GH, FT3, TT, and PRL levels are correlated with POD1GH levels, with some variations observed between the sexes. Preoperative GH levels of ≥30.25 ng/mL, preoperative FT3 levels of ≤4.415 pmol/L, and age of ≤51 years are predictive of POD1GH level non-remission. Therefore, predicting postoperative efficacy requires comprehensive consideration of the status of multiple hormonal axes. Hormone levels measured using precision instruments can serve as an adjunct to imaging-based tumor characteristics for predicting postoperative somatotropic axis function.

## Limitations

This study has certain limitations. First, it is a single-center, retrospective study, and the sample size is limited due to the rarity of acromegaly. Second, the sex hormones of non-menopausal female patients fluctuate with the menstrual cycle. Given the exploratory nature of this study, clear distinctions were not made between premenopausal and menopausal women in the regression analysis. In addition, to control confounding factors, patients who underwent endoscopic transsphenoidal surgery were not included in the screening process, and the conclusions may not be applicable to endoscopic approaches. Lastly, this study primarily discusses the correlation between perioperative hormone levels and does not analyze the final outcomes of each hormone change. Further research is necessary to explore whether such associations among hormones persist in the long term in patients.

## Abbreviations

ACTH: adrenocorticotropic hormone
AUC: area under the curve
COR: cortisol
E2: estradiol
FSH: follicle stimulating hormone
FT3: free triiodothyronine
FT4: free thyroxine
GH: growth hormone
GTR: gross total resection
HPA: hypothalamic-pituitary-adrenal
HPG: hypothalamic-pituitary-gonadal
HPT: hypothalamic-pituitary-thyroid
IGF-1: insulin-like growth factor-1
LH: luteinizing hormone
mTSS: microscopic transsphenoidal surgery
PA: pituitary adenoma
POD1: postoperative day one
PRL: prolactin
ROC: receiver operating characteristic
STR: subtotal resection
TSH: thyroid stimulating hormone
TT: total testosterone
TT3: total triiodothyronine
TT4: total thyroxine
